# Development of a Virtual Chinese Pediatric Population Physiological Model Targeting Specific Metabolism and Kidney Elimination Pathways

**DOI:** 10.3389/fphar.2021.648697

**Published:** 2021-05-11

**Authors:** Xueting Yao, Xuanlin Liu, Siqi Tu, Xiaobei Li, Zihan Lei, Zhe Hou, Zhiheng Yu, Cheng Cui, Zhongqi Dong, Farzaneh Salem, Haiyan Li, Dongyang Liu

**Affiliations:** ^1^ Drug Clinical Trial Center, Peking University Third Hospital, Beijing, China; ^2^ School of Pharmaceutical Sciences, Tsinghua University, Beijing, China; ^3^ School of Pharmaceutical Sciences, Peking University Health Science Center, Peking University, Beijing, China; ^4^ School of Basic Medicine and Clinical Pharmacy, China Pharmaceutical University, Nanjing, China; ^5^ Janssen China R&D Center, Shanghai, China; ^6^ Certara UK Limited, Simcyp Division, Sheffield, United Kingdom; ^7^ Department of Cardiology and Institute of Vascular Medicine, Peking University Third Hospital, Beijing, China

**Keywords:** PBPK, Chinese pediatric population, CYP1A2, CYP3A4, renal elimination, pharmacokinetics

## Abstract

**Background:** Physiologically based pharmacokinetic (PBPK) modeling and simulating may be a powerful tool in predicting drug behaviors in specific populations. It is a mathematical model that relates the pharmacokinetic (PK) profile of a compound with human anatomical characteristics, physiological characteristics, and biochemical parameters. Predictions using PBPK models offer a promising way to guide drug development and can be used to optimize clinical dosing regimens. However, PK data of new drugs in the pediatric population are too limited to guide clinical therapy, which may lead to frequent adverse events or insufficient efficacy for pediatric patients, particularly in neonates and infants.

**Objective:** The objective of this study was to establish a virtual Chinese pediatric population based on the physiological parameters of Chinese children that could be utilized in PBPK models.

**Methods:** A Chinese pediatric PBPK model was developed in Simcyp Simulator by collecting published Chinese pediatric physiological and anthropometric data to use as system parameters. This pediatric population model was then evaluated in the Chinese pediatric population by predicting the pharmacokinetic characteristics of four probe drugs: theophylline (major CYP1A2 substrate), fentanyl (major CYP3A4 substrate), vancomycin, and ceftazidime (renal-eliminated).

**Results:** The predicted maximum concentration (C_max_), area under the curve of concentration-time (AUC), and clearance (CL) for theophylline (CYP1A2 metabolism pathway) and fentanyl (CYP3A4 metabolism pathway) were within two folds of the observed data. For drugs mainly eliminated by renal clearance (vancomycin and ceftazidime) in the Chinese pediatric population, the ratio of prediction to observation for major PK parameters was within a 2-fold error range.

**Conclusion:** The model is a supplement to the previous Chinese population PBPK model. We anticipate the model to be a better representative of the pediatric Chinese population for drugs PK, offering greater clinical precision for medication given to the pediatric population, ultimately advancing clinical development of pediatric drugs. We can refine this model further by collecting more physiological parameters of Chinese children.

## Highlights

Pediatric rational medication and drug development have received much attention these years. However, the *in vivo* PK data are limited in the Chinese pediatric population, which is an obstacle in clinical trials to guide dosing regimen. In this study, we developed a Chinese pediatric PBPK model to predict *in vivo* drug behavior in the Chinese pediatric population. We anticipate the model to assist in pediatric drug development and rational clinical medication.

## Introduction

The rate of adverse drug reactions (ADR) reported in Chinese children and newborns are two and four times higher than that of adults (NMPA, 2018). One of the key reasons for the high adverse events (AE) rate is the irrational dosing regimen found in the pediatric population. On one hand, evidence-based clinical trials are the gold-standard for the optimization of dosing regimens, which are generally missing during pediatric drug development. On the other hand, dose regimens for children are usually extrapolated from adult populations based on body weight. In some cases this extrapolation cannot be applied to pediatrics because such an approach assumes that all metabolic enzymes and transporters mature relative to body weight, which is not necessarily true. Thus in 2017, the China National Medical Products Administration (NMPA) released regulatory guidelines that required evidence-based pharmacokinetic and pharmacodynamics (PK/PD) data to support pediatric drug development (NMPA, 2017). However, it is difficult to conduct dedicated clinical trials to observe children’s PK data due to ethical reasons.

The physiologically based pharmacokinetic (PBPK) model could integrate drug and physiological information and clinical trial design to make predictions on PK in special populations, including pediatrics. For example, Abduljalil et al. developed a preterm PBPK model by collecting published information on preterm developmental physiology including demographics, hematocrit, protein binding data, tissue volume, and enzyme ontogeny data (Abduljalil et al., 2019a). The model was then evaluated in compounds metabolized by CYP1A2, CYP2C9, and CYP3A4 as well as two renal-eliminated antibiotics. The model proved reliable in predicting PK behavior in Caucasian preterm neonates. Besides, Barrett et al. have also summarized PBPK modeling and its application to assist in pediatric pharmacology studies and study design (Barrett et al., 2012). Although the PBPK approach has been frequently applied to support drug development in western countries, it is rarely utilized in China. One of the main limitations of using PBPK in the Chinese pediatric population is the lack of the physiological parameters and ontogeny data of Chinese pediatric population, which may not directly adopt the Caucasian pediatric population model due to genetic differences between distinct ethnic groups. PK differences between Caucasian and Chinese children were reported by Gao et al. and Song et al. for caffeine (Gao et al., 2020) and vancomycin (Song et al., 2017). The authors concluded that these differences could be attributed to a higher drug clearance in Chinese children than in Caucasian children. The ethnic differences may come from enzyme or transporter ontogeny, they could also come from the growth chart of body size or tissue volume. However, whether there are ethnic differences between Chinese and Caucasian children still needs to be further verified by clinical studies or mechanic method such as PBPK modeling.

Therefore, the objective of this study is to develop a Chinese pediatric population model by incorporating the physiological parameters specific to the Chinese pediatric population with reference to the Caucasian population model. The new Chinese pediatric population model will be used in Simcyp Simulator to predict PK for CYP1A2 probe substrate (theophylline), CYP3A4 probe substrate (fentanyl) and renal-eliminated drugs (vancomycin and ceftazidime) in Chinese *in vivo* PK data. We anticipate that this model can assist in Chinese pediatric drug development and rational clinical medication.

## Methods

### General Workflow

**FIGURE 1:**
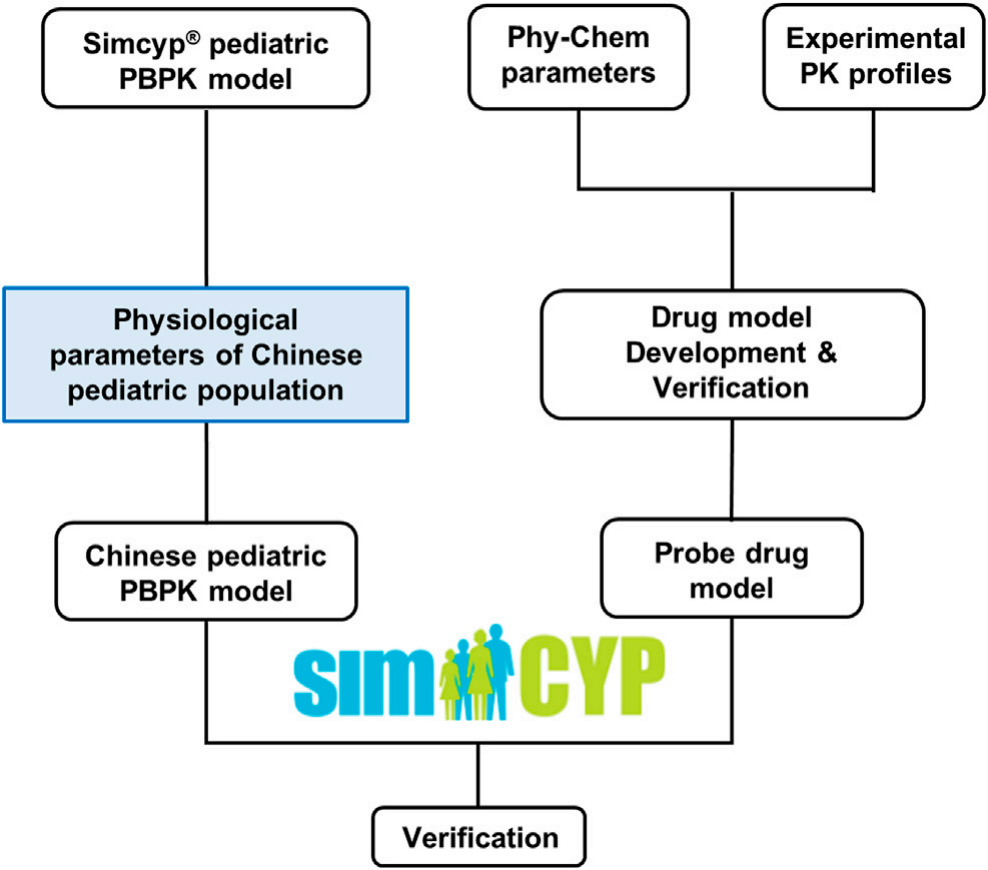
The general workflow.

Figure 1 illustrated the overall study strategy. Briefly, physiological and demographic data of Chinese children aged from 0 to 18 years old were first collected. These data were then used to recalibrate the corresponding physiological parameters in the Caucasian pediatric population model in Simcyp^®^ (Version 20, Simcyp Division, Certara UK Limited, United Kingdom) to generate a Chinese pediatric population model. Next, a literature search for clinical PK data in the Chinese pediatric population was carried out with specific inclusion and exclusion criteria. Based on the availability of the PK data and specificity of the substrate, four probe drugs, predominantly eliminated by three different pathways (CYP1A2, CYP3A4 and renal-filtration), were selected. Drug models were then developed for these probe drugs in Simcyp and were verified using PK data from the Caucasian adults, Caucasian children, and Chinese adults. Finally, the drug models of these probe drugs, in combination with the newly developed Chinese pediatric population model, were applied to simulate PK in the Chinese pediatric population to evaluate the population model. The prediction performance of the models was evaluated by comparing the simulated maximum concentration (C_max_), clearance (CL), and area under the concentration-time curve (AUC) with the observed value.

### Development of Chinese Pediatric Population Model

#### Physiological and Demographic Data Collection

A systematic literature search was carried out for various physiological and demographic data of Chinese children using PubMed (https://www.ncbi.nlm.nih.gov/pubmed/). Keywords used in the search included “Chinese” in all combinations with “demographic”, “height”, “weight”, “organ weight (brain, heart, kidney, liver, lung, pancreas, spleen, thyroid gland, adrenal gland)”, “cardiac output”, “body surface area (BSA)”, “yellow marrow and blood volume”, “tissue compositions (tissue weight/volume, tissue water volume, lipid weight/volume)”, “glomerular filtration rate (GFR)”, “biologic parameters (IgG, IgE, FcRn, TNF-alfa, lymph volume, lymph flow rate)”, “ontogeny (cytochrome P450, glucuronyltransferase, transporters, flavoprotein monooxygenases, carboxylesterases)”, and “skin parameters (total skin thickness, skin surface pH, stratum corneum thickness, dermis thickness, viable epidermis thickness)”. Data were included in our study if they met the following criteria: 1) the data came from Chinese children aged 0–18 years old; 2) male and female statistics were separate (in specific parameters).

#### Population Model Development

The Chinese pediatric population model was developed in Simcyp based on the inbuilt Northern European Caucasian (NEC) pediatric population model. The physiological and demographic data in the Chinese pediatric population collected and included in our study were used to recalibrate the corresponding parameters in the NEC pediatric population model. All the parameters were modeled against age, height, weight, or BSA using inbuilt equations of the NEC pediatric population. By integrating the equations of physiological and anthropometric parameters into the Simcyp simulator, the equations of height and weight were set as inputs in “Population-Demographic-User-defined HT and WT relationship”. Equations of organ weight were modified in “Population-Paediatric-Tissue Volume-Define Tissue Volume”. The cardiac output function was defined in “Population-Paediatric-Blood Flows-Define Blood Flow”. For the rest of the physiological parameters, because there was very limited Chinese pediatric data available, the default value of the inbuilt NEC pediatric population model was used with the assumption that they were the same between Chinese and Caucasians.

### Development of Drugs Model for Probe Drugs

#### Clinical Pharmacokinetic Data Collection and Probe Drug Selection

**FIGURE 2:**
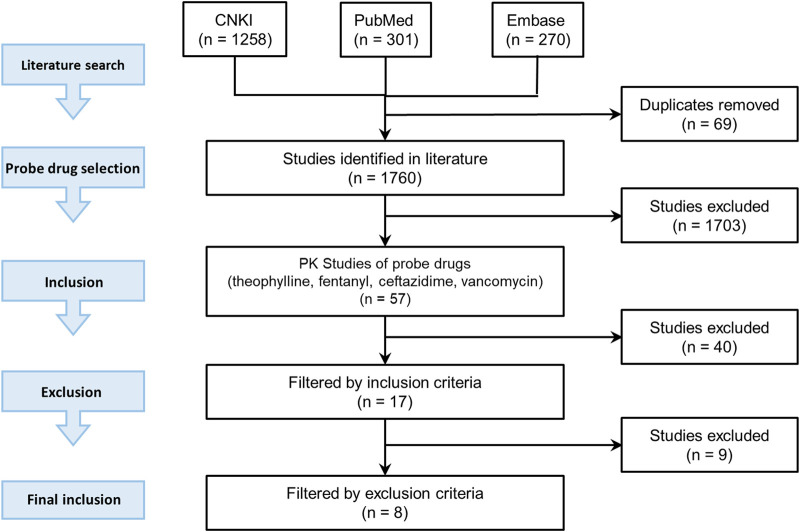
Demonstration of literature search strategy.

The candidate probe drugs were selected based on the following considerations: a sensitive substrate of CYP1A2 or CYP3A4, namely more than 80% of the drug was metabolized and more than 70% metabolism was contributed by CYP1A2 or CYP3A4; or more than 70% of the drug was eliminated by renal filtration. A literature search was then conducted to collect PK data of candidate probe drugs in the Chinese pediatrics population at different ages using PubMed (https://www.ncbi.nlm.nih.gov/pubmed/), Embase (https://www.embase.com), and CNKI (https://www.cnki.net/). The literature search strategy was illustrated in Figure 2.

Three keywords were used in the search: “pharmacokinetics”, keywords related to different age groupings of pediatrics (e.g., “pediatrics”, “children”, “infants”, “neonates”), and the keyword related to the targeted race and region (e.g., “Chinese”, “China”). Deadline for inclusion in the literature is 2019. The related drugs were included in the probe drug candidate list.

Among the drugs with PK data included in our study, four probe drugs (i.e., theophylline, fentanyl, vancomycin, and ceftazidime) that were mainly eliminated by three respective pathways (CYP1A2 pathway, CYP3A4 pathway and renal-eliminated pathway) were selected. Theophylline is one of the substrate drugs of CYP1A2, and CYP1A2 contributed 75% of its metabolism (Wu and Peters, 2019). Theophylline metabolism was used as a probe to investigate CYP1A2 activity *in vivo* (Wang et al., 2013). Fentanyl is converted to a major metabolite, namely norfentanyl, in human liver by CYP3A4-mediated pathway (Feierman and Lasker, 1996). Ceftazidime and vancomycin are mainly eliminated through renal filtration, which accounted for 70–80% of their elimination (Welage et al., 1984; DrugBank). The clearance of ceftazidime and vancomycin was used to indicate the glomerular filtration function.

There were 57 studies related to these four probe drugs. If the study met the following inclusion criteria but not exclusion criteria, the PK data such as C_max_, CL, AUC, and the concentration-time profile were included in our study. Inclusion criteria were: 1) study subjects were of the Chinese pediatric population, included neonates, infants, children, and adolescents with normal cardiac, hepatic and renal functions 2) sample size was larger than three 3) dosing regimen was reported 4) the drug was administered intravenously 5) there was no comedication in the study and 6) drug concentration-time profiles or at least one of the PK parameter was reported. Exclusion criteria were: 1) participants included preterm infants 2) the age range of subjects was not reported clearly 3) dosing regimen varied from individual to individual and the mean dosage was not reported.

Seventeen studies were included according to inclusion criteria and nine studies were excluded. Four of them were excluded for meeting criteria one, two of them were excluded for criteria two, and three of them were excluded for criteria three. Finally, eight studies met our criteria and were included in our research.

#### Development and Verification of Drugs Model for Probe Drugs

Theophylline compound file from Simcyp V20 library was used to carry out simulations. Fentanyl, ceftazidime and vancomycin PBPK models were self-developed compound files. The physicochemical properties, as well as the drug metabolism and pharmacokinetics (DMPK) characteristics of the probe drugs that need to be self-developed, were obtained from the Drug Bank (https://www.drugbank.ca/), SciFinder (https://scifinder.cas.org), and literature. The collected data were then integrated into a PBPK model using Simcyp Simulator.

A minimal distribution model was applied in theophylline drug model due to better performance in PK prediction, compared to full PBPK model. On the other hand, the full PBPK model was used in fentanyl, ceftazidime and vancomycin. As for absorption model, the first-order absorption model was used if the drug was administrated intravenously while the advanced dissolution, absorption and metabolism (ADAM) model was used in oral administration cases. The PBPK model parameters of probe drugs were presented in Table 1.

**TABLE 1:** Summary of PBPK model parameters of probe drugs.

Parameter	Theophylline	References	Fentanyl	References	Ceftazidime	References	Vancomycin	References
MW (g/mol)	180.2	Simcyp	336.47	Drugbank	546.58	Scifinder	1449.26	Scifinder
Log P	−0.02	Simcyp	4.05	PubChem	-2.65	Scifinder	−3.75	PubChem
Compound type	Ampholyte	Simcyp	Base	—	Diprotic acid	–	Ampholyte	—
pK_a_1	8.8	Simcyp	8.99	Drugbank	2.4	Scifinder	2.18	FDA NDA review
pK_a_2	0.99	Simcyp	—	—	4.26	Drugbank	7.75	FDA NDA review
B/P	0.82	Simcyp	0.87	Jantos et al. (2011)	0.55	Cui et al. (2020b)	0.75	Abduljalil et al. (2019b)
f_u_	0.5/0.62^a^	Simcyp, Bjorkman (2005)	0.16	Ji et al. (2019)	0.9	FDA label	0.672	Abduljalil et al. (2019b)
Distribution model	Minimal PBPK model	Simcyp	Full PBPK model (perfusion-rate-limited)	Ji et al. (2019)	Full PBPK model (perfusion-rate-limited)	—	Full PBPK model (perfusion-rate-limited)	—
Major elimination pathway	Metabolism (90%) Renal (10%)	Wu and Peters (2019)	Metabolism (94%) Renal (6%)	Encinas et al. (2013)	Renal (73%)	Welage et al. (1984)	Renal (75–80%)	Drugbank
Fractional contribution of enzyme	CYP1A2 (75%)	Simcyp	CYP3A4 (100%)	Feierman and Lasker (1996), Labroo et al. (1997), Encinas et al. (2013)	_	–	_	–
	CYP2D6 (7%)							
	CYP2E1 (10%)							
	CYP3A4 (8%)							
*In vitro* intrinsic clearance (CL_int_, μL/min/pmol P450)	CL_int_ 1A2 = 0.02	Simcyp	CL_int_ 3A4 = 0.707	Ji et al. (2019)	—	—	—	—
	CL_int_ 2D6 = 0.011							
	CL_int_ 2E1 = 0.0022							
	CL_int_ 3A4 = 0.00078							
CL_R_ (L/h)	0.31	Simcyp	2.232	Encinas et al. (2013)	6	Cui et al. (2020a)	6	Abduljalil et al. (2019b)
V_ss_ (L/kg)	0.5/0.6^a^	Ogilvie (1978), Bjorkman (2005)	4.071	Predicted^b^	0.195	Predicted^b^	0.445	Predicted^b^

MW, molecular weight; B/P, blood to plasma partition ratio; f_u_, fraction unbound; CL_R_, renal clearance; V_ss_, volume of distribution at steady state.

^a^ The value applied to simulations in neonates.

^b^ Predicted value using Simcyp.

The drug models for all four probe drugs were verified in a stepwise manner using clinical PK data from Caucasian adults, Caucasian children, and Chinese adults. The major PK parameters (C_max_, CL, AUC) and the concentration-time profile were included in drug model verification if the study met the following inclusion criteria: 1) study subjects were of the Chinese/Caucasian adult population or the Caucasian pediatric population with normal cardiac, hepatic and renal functions; 2) sample size was larger than three; 3) dosing regimen was reported; 4) there was no comedication in the study and 5) drug concentration-time profiles or at least one of the PK parameters was reported. If the ratio of predicted and observed mean values for major PK parameters was within the range of 0.5–2.0, these drug models were deemed fit to be verified.

### Evaluation of Chinese Pediatric Population Models

The verified drug models of four probe drugs in combination with the newly developed Chinese pediatric population model were applied to predict drug PK in the Chinese pediatric population. The simulation was conducted by mimicking trial designs in the literature. The predicted drug concentrations vs. the observed ones were examined. Furthermore, the predicted PK parameters (i.e., C_max_, CL, and AUC) of each probe drug were compared with the observed values. If the ratio of the predicted value was within a 2 folds difference to the observed one, the population model was considered acceptable.

## Results

### Chinese Pediatric Population Model Development

Body height, weight, cardiac output, albumin concentration, hematocrit and organ weight (brain, heart, kidney, liver, pancreas, spleen) in the Chinese pediatric population were collected in our study but there is still a lack of information on enzyme and transporter’s ontogeny, GFR, some of the tissue volumes (lung, blood), tissue compositions, biologic parameters (i.e., IgE, FcRn, TNF-alfa, lymph volume, lymph flow rate, etc.), and skin parameters (i.e., total skin thickness, skin surface pH, etc.).

The height and weight data were collected from two pieces of literature covering Chinese children aged 0–18 years old (Janssen et al., 2007; Zong and Li, 2013). The cardiac output data were collected from children aged from 1 to 12 years old (Cattermole et al., 2017). All the organ weight data were obtained from children aged from 0 to 15 years old (Wang et al., 1995a; Kawamura, 1998). These data were then used to develop the Chinese pediatric population model by recalibrating the equations for describing the change of these physiological characteristics with age in the NEC pediatric population model. As shown in Table 2, the regression curves fit the observation data well with regression coefficient (*R* ^2^) > 0.98. Table 3 showed some anthropometry data (height, weight, BMI, and heart rate), blood biochemistry indexes (serum creatinine concentration and albumin concentration) and hematocrit in Chinese children from national physique and health database. They were used to verify the performance of Chinese pediatric model in predicting these parameters.

**TABLE 2:** Physiological parameter equations of Chinese pediatric population.

Physiological parameters	Male	Female
Height (HT)	0.00002161283469646225 × Age^7^ − 0.001402674299655576 × Age^6^ + 0.03620932170728922 × Age^5^ − 0.4768902367461678 × Age^4^ + 3.430362938724804 × Age^3^ − 13.55291585347404 × Age^2^ + 34.65861528448144 × Age + 52.91366922873476 (*R* ^2^ = 0.99)	− 0.000003237786173327683 × Age^8^ + 0.0002451527760663450 × Age^7^ − 0.007615458476879155 × Age^6^ + 0.1254101134395548 × Age^5^ − 1.184629069972546 × Age^4^ + 6.513533858016925 × Age^3^ − 20.44446259722061 × Age^2^ + 41.44107707509581 × Age + 49.589645987915 (*R* ^2^ = 0.99)
Weight (WT)	$4.665\times \left[1-{\text{e}}^{\left(-1.661\right)\times \text{Age}}\right]+{\text{e}}^{\left(0.025\times \text{HT}\right)+\left(-0.015\times \text{Age}\right)}$ (*R* ^2^ = 0.99)	$5.087\;\times \left[1-{\text{e}}^{\left(-1.793\right)\times \text{Age}}\right]+{\text{e}}^{\left(0.023\times \text{HT}\right)+\left(0.012\times \text{Age}\right)}$ (*R* ^2^ = 1)
Cardiac output	$\text{BSA}\times \left[197.444+126.667\times \left({\text{e}}^{-0.06\times \text{Age}}-{\text{e}}^{-0.774\times \text{Age}}\right)\right]$ (*R* ^2^ = 0.99)	
Brain volume	0.473×WT−0.08754×WT×ln(HT) (*R* ^2^ = 0.98)	0.483548×WT−0.090402×WT×ln(HT) (*R* ^2^ = 0.98)
Heart volume	$\frac{0.13\times {\text{BSA}}^{1.26}}{1.04}$ (*R* ^2^ = 0.99)	$\frac{0.1277\times {\text{BSA}}^{1.3055}}{1.04}$ (*R* ^2^ = 0.99)
Kidney volume	$\frac{13.028\times {\text{WT}}^{0.713}}{1000}$ (*R* ^2^ = 0.99)	$\frac{5.509\times {\text{WT}}^{0.761}+5.837\times {\text{WT}}^{0.762}}{1000}$ (*R* ^2^ = 0.99)
Liver volume	$0.679\times {\text{BSA}}^{1.0345}$ (*R* ^2^ = 0.99)	$0.695\times {\text{BSA}}^{1.125}$ (*R* ^2^ = 0.99)
Pancreas volume	$\frac{{\text{e}}^{-3.5+1.997\times \ln\left(\frac{\text{HT}}{100}\right)}}{1.05}$ (*R* ^2^ = 0.99)	$\frac{{\text{e}}^{-3.549+2.251\times \ln\left(\frac{\text{HT}}{100}\right)}}{1.05}$ (*R* ^2^ = 0.98)
Spleen volume	$\frac{0.023\times \left(\frac{\text{HT}}{100}\right)\times {\text{WT}}^{0.351}}{1.06}$ (*R* ^2^ = 0.99)	$\frac{0.017\times \left(\frac{\text{HT}}{100}\right)\times {\text{WT}}^{0.41}}{1.06}$ (*R* ^2^ = 0.99)

BSA for under 15 kg subjects is calculated from Haycock et al., 1978 (BSA = WT^0.5378^ × HT^0.3964^ × 0.024265) and for subjects equal to or greater than 15 kg is calculated from Du Bois and Du Bois 1916. (BSA = WT^0.425^ × HT^0.725^ × 0.007246).

**TABLE 3:** Summary of anthropometry data and hematological indexes in Chinese pediatric population^a^.

Parameters	Less than 11 years		11–12 years		13–14 years		15–16 years		17–18 years	
Gender	Male	Female	Male	Female	Male	Female	Male	Female	Male	Female
*N*	1660	1798	3577	3522	3696	3854	3675	4688	2322	2894
Height (cm)	138.72 ± 8.25 [122.40–155.00]	138.64 ± 8.26 [122.10–155.00]	147.67 ± 9.15 [131.04–167.00]	148.56 ± 7.96 [132.31–163.00]	159.31 ± 9.49 [139.89–176.00]	155.27 ± 6.45 [142.00–167.76]	167.76 ± 7.38 [152.00–181.00]	157.70 ± 5.80 [146.40–169.00]	169.78 ± 6.22 [157.00–181.99]	157.66 ± 5.45 [147.00–169.00]
*N*	1660	1800	3583	3532	3701	3856	3679	4696	2322	2897
Weight (kg)	34.03 ± 8.46 [23.05–56.89]	32.68 ± 7.51 [22.20–51.99]	40.60 ± 10.59 [26.70–67.08]	40.32 ± 9.04 [26.20–62.00]	49.38 ± 11.73 [31.76–79.22]	47.37 ± 8.12 [33.40–66.50]	57.28 ± 11.61 [40.50–88.40]	51.62 ± 7.69 [40.00–70.00]	59.43 ± 9.43 [45.10–83.50]	51.97 ± 7.00 [41.00–68.50]
*N*	1657	1798	3576	3522	3695	3853	3675	4688	2322	2894
BMI (kg/m^2^)	17.50 ± 3.02 [13.71–25.49]	16.84 ± 2.56 [13.34–23.73]	18.40 ± 3.31 [14.01–26.59]	18.11 ± 2.93 [13.84–25.41]	19.27 ± 3.25 [15.05–28.33]	19.58 ± 2.74 [15.19–26.34]	20.27 ± 3.38 [15.94–29.91]	20.74 ± 2.74 [16.40–27.11]	20.58 ± 2.81 [16.65–27.91]	20.90 ± 2.54 [16.79–26.88]
*N*	1612	1771	3524	3466	3662	3819	3639	4658	2281	2864
Heart rate (beats/min)	90.58 ± 14.25 [64.00–123.00]	94.45 ± 14.28 [68.00–125.00]	90.36 ± 14.36 [65.00–120.00]	94.48 ± 14.99 [68.00–127.00]	84.00 ± 13.40 [60.58–113.00]	89.38 ± 13.99 [66.00–121.00]	78.94 ± 13.42 [56.00–109.00]	85.31 ± 13.49 [62.00–115.00]	75.76 ± 14.30 [51.00–109.00]	83.88 ± 13.33 [62.00–115.00]
*N*	592	589	1426	1434	1718	1756	1672	1897	1058	1031
Serum creatine (μmol/L)	52.11 ± 11.49 [35.00–77.00]	50.19 ± 11.75 [33.00–75.25]	56.62 ± 11.91 [37.00–79.00]	52.56 ± 11.30 [34.00–75.00]	62.32 ± 14.03 [39.00–93.00]	55.84 ± 12.27 [37.00–82.00]	73.34 ± 13.80 [48.00–102.00]	60.85 ± 12.15 [41.00–85.00]	77.77 ± 13.07 [55.00–105.00]	60.43 ± 12.12 [40.80–84.00]
*N*	592	589	1427	1433	1718	1753	1671	1891	1057	1030
Serum albumin (g/L)	47.96 ± 2.66 [43.10–53.22]	47.65 ± 2.62 [43.00–53.58]	47.86 ± 2.89 [42.77–54.00]	47.90 ± 2.86 [42.90–54.10]	48.20 ± 3.21 [42.30–55.00]	48.29 ± 3.17 [42.38 ± 55.10]	49.25 ± 3.29 [43.48–56.20]	48.94 ± 3.26 [43.10–56.27]	49.88 ± 3.11 [44.00–56.56]	48.89 ± 3.07 [43.30–55.50]
*N*	1377	1488	3040	2973	2962	3195	3300	4161	2030	2548
Hematocrit (%)	40.85 ± 2.44 [36.44–46.00]	40.97 ± 2.42 [36.20–45.80]	41.96 ± 2.58 [37.00–47.30]	41.83 ± 2.47 [37.00–46.50]	43.87 ± 2.86 [38.10–49.50]	41.95 ± 2.72 [36.39–47.20]	46.02 ± 2.94 [39.50–51.40]	41.94 ± 2.68 [36.10–46.80]	46.84 ± 2.90 [40.90–52.20]	41.73 ± 2.64 [36.20–46.70]

Data from National Physique and Health Database (2006–2011).

^a^ Data were presented as mean ± SD [2.5th percentile–97.5th percentile].

**FIGURE 3:**
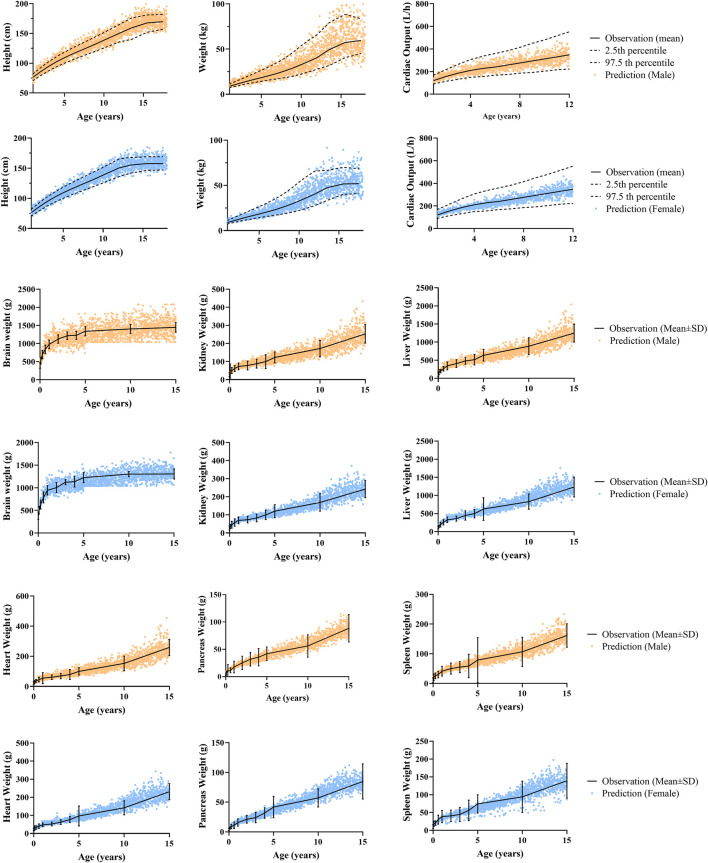
Predicted and observed physiological data in male and female. Anthropometric data (height, weight) and cardiac output data aged from 1 to 12 years old were reported by Cattermole et al. (2017), and anthropometric data aged from 13 to 18 years old were from the National Physique and Health Database (http://cnphd.bmicc.cn/chs/en/index.php). Organ weight data aged from 0 to 15 years old were reported by Wang at al. (1995a).

To evaluate the capability of the developed pediatric population model to describe the ontogeny of height, weight, cardiac output and organ size of Chinese children, a simulation of 4000 virtual Chinese pediatric population was performed using Simcyp simulator (proportion of females = 0.5; 200 subjects in each trial). As shown in Figure 3, most of the simulated height, weight, and cardiac output in Chinese children were within the 2.5th and the 97.5th percentile of observation for both male and female, and the organ weights were evenly distributed around the observed mean value, indicating that the newly developed Chinese pediatric population model can describe the ontogeny of body size, cardiac output and certain tissue volume of Chinese children.

### Probe Drugs Physiologically Based Pharmacokinetic Model Development and Verification

The drug model for all four probe drugs was verified with PK data of Caucasian adults, the Caucasian pediatric population, and the Chinese adult population from various studies. As shown in Supplementary Table S2 and Supplementary Figure S1, 90.48% of the predicted PK parameters of theophylline drug model were within the 2-fold error range, and 57.14% of them were within the 1.25 fold error range. The corresponding values of fentanyl, ceftazidime and vancomycin drug models were 84.85% and 30.30%, 92.86% and 78.57%, and 92.31% and 61.54%, respectively. In conclusion, over 89.47% of the prediction to observation (P/O) ratios were between 0.5 and 2.0 in all the established probe drug models, indicating the consistency and robustness of our probe drug model. For drug concentration-time profiles, most of the observed plasma concentrations were within the 5th to the 95th percentile of prediction (Supplementary Figure S2).

### Chinese Pediatric Population Model Verification

**FIGURE 4:**
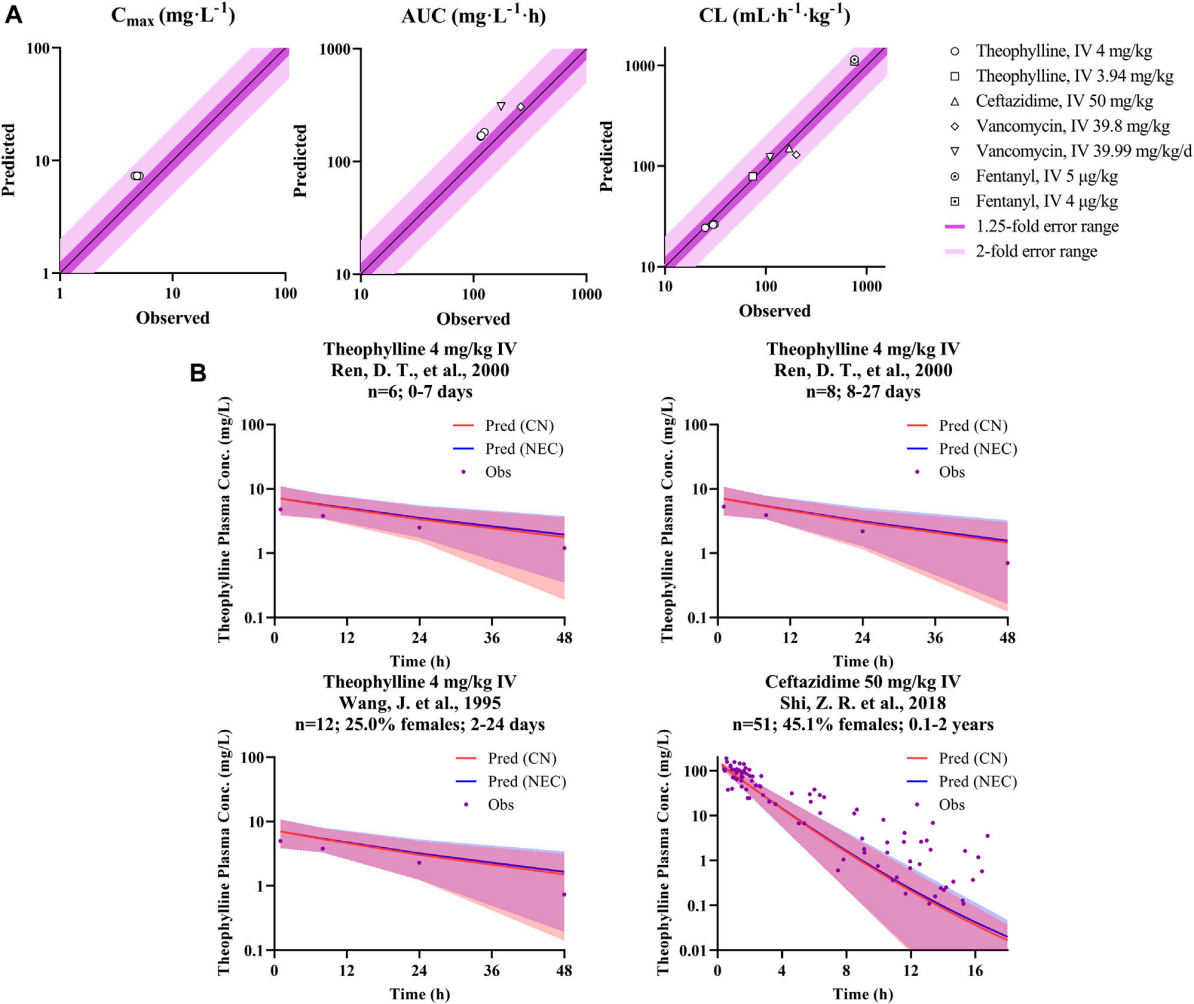
P/O ratio of PK parameters and drug concentration-time profiles. **(A)** The observed PK parameter (C_max_, CL, and AUC) vs. predicted values of probe drugs using developed Chinese pediatric population model. **(B)** The observed and predicted concentration data of probe drugs using developed Chinese pediatric population model. (Dots represents observed data; blue line represents predicted data based on Caucasian pediatric model; red line represents predicted data based on Chinese pediatric model; and blue/red shadow represents 90% prediction interval of the corresponding model, purple shadow is their overlapped parts).

The verified drug model of these four probe drugs in combination with the developed Chinese pediatric population model was applied to predict drug concentration in the Chinese pediatric population. As shown in Table 4 and Figure 4A, for theophylline (major CYP1A2 substrate) and fentanyl (major CYP3A4 substrate), ratios of predicted vs. observed PK parameters (C_max_, CL, AUC) from various studies were within the range of 0.5–2.0. For ceftazidime and vancomycin, which were eliminated via renal elimination pathway, the ratio of predicted vs. observed CL from the single study of ceftazidime was 0.88, and the ratio of vacomycin was 1.12 and 0.65. The ratio of predicted vs. observed AUC for vancomycin was 1.76 and 1.17, respectively. No C_max_ was reported for both drugs. Figure 4B also illustrated the observed and predicted concentration-time profile for theophylline and ceftazidime. Compared to the concentration-time profile predicted in NEC pediatric model, the mean concentration decreased slightly in Chinese pediatric model and most of the observed plasma concentrations were within the 5th and 95th percentile of prediction. The concentration-time profiles of fentanyl and vancomycin were not reported in children with normal cardiac, hepatic and renal function.

**TABLE 4:** PK parameters of probe drugs predicted by CN pediatric model in Chinese pediatric population.

Drug	References	Dose	Age (years)	PK parameter	Chinese pediatric model				
					Predicted	SD	Observed	SD	Ratio
Theophylline	Ren et al. (2000)	4 mg/kg IV	0–0.02	C_max_ (mg/L)	7.31	2.26	4.62	NA	1.58
				CL (mL/h·kg)	24.39	8.60	25.00	6.00	0.98
				AUC_0-t_ (mg h/L)	181.72	56.11	124.90^a^	NA	1.45
Theophylline	Ren et al. (2000)	4 mg/kg IV	0.02–0.07	C_max_ (mg/L)	7.30	2.26	5.07	NA	1.44
				CL (ml/h kg)	26.53	10.18	31.00	5.00	0.86
				AUC_0-t_ (mg h/L)	167.08	51.33	115.8^a^	NA	1.44
Theophylline	Chen (1989)	3.94 mg/kg IV	6.6–12.25	C_max_ (mg/L)	8.51	2.58	NA	NA	NA
				CL (ml/h·kg)	78.90	30.95	74.00	20.00	1.07
				AUC_0-t_ (mg h/L)	54.38	16.86	NA	NA	NA
Theophylline	Wang et al. (1995b)	4 mg/kg IV	0–0.07	C_max_ (mg/L)	7.31	2.25	4.83	NA	1.51
				CL (ml/h kg)	26.08	7.56	30.00	5.00	0.87
				AUC_0-t_ (mg h/L)	169.61	52.27	116.8^a^	NA	1.45
Fentanyl	Xu et al. (2001)	5 μg/kg IV	8–13	C_max_ (ng/ml)	22.73	2.41	NA	NA	NA
				CL (ml/h kg)	1146.71	248.98	756.00	168.00	1.52
				AUC_0-t_ (ng h/ml)	4.35	0.61	NA	NA	NA
Fentanyl	Li et al. (2013)	4 μg/kg IV	6–16	C_max_ (ng/ml)	18.81	2.74	NA	NA	NA
				CL (ml/h kg)	1096.58	346.95	756.00	168.80	1.45
				AUC_0-t_ (ng h/ml)	3.62	0.55	NA	NA	NA
Ceftazidime	Shi et al. (2018)	50 mg/kg IV	0.1–2	C_max_ (mg/L)	188.38	9.70	NA	NA	NA
				CL (ml/h kg)	150.17	48.64	170.00	NA	0.88
				AUC_0-t_ (mg h/L)	346.06	64.27	NA	NA	NA
Vancomycin	Jiang et al. (2015)	39.99 mg/kg/d IV	0.08–14	C_max_ (mg/L)	29.57	3.27	NA	NA	NA
				CL (ml/h kg)	122.47	53.59	109.74	43.68	1.12
				AUC_0–24h_ (mg h/L)	307.99	57.96	175.34	157.86	1.76
Vancomycin	Zhang et al. (2016)	39.8 mg/kg IV	0.09–2	C_max_ (mg/L)	31.65	2.72	NA	NA	NA
				CL (ml/h kg)	129.65	45.68	200.00	100.00	0.65
				AUC_0–24h_ (mg h/L)	306.15	57.15	261.4	105.10	1.17

NA, not applicable.

^a^ Calculated using trapezoidal rule from concentration-time profiles in literature.

## Discussion

In the past decades, the PBPK model has been applied to predict pediatric drug concentrations during drug development to support dosing rationalization in the pediatric clinical trial. For example, the PBPK model was used to set a starting dose for Eribulin in children and adolescents aged 6–18 years old (Shebley et al., 2018). However, majority of its application is in Caucasian pediatrics with very limited examples to apply the PBPK model in other ethnic groups. Recently, Kim et al. developed a PBPK model for the Korean pediatric population in Simcyp. The model was verified using six substrate drugs of five major metabolic enzymes and two transporters. The model was proved useful in predicting the concentration-time profiles of these drugs (Kim et al., 2019). In our study, a similar approach was adopted to develop the Chinese pediatric population model by incorporating Chinese pediatric demographic and physiological information covering a span of children 0–15 years old. Our study, with limited examples, showed that the developed pediatric population model can achieve reasonable predictions on drug exposure in the Chinese pediatric population. This may serve as the first step in developing a more mature Chinese pediatric population model in the future.

In our study, depending on the availability of the Chinese pediatric PK data, four probe drugs that were eliminated either by CYP1A2 metabolism (theophylline), CYP3A4 metabolism (fentanyl) or by renal filtration (ceftazidime and vancomycin) were selected to evaluate the prediction performance of this pediatric population model. It should be noted that CYP1A2 and CYP3A4 are the major P450 enzyme in eliminating several drugs in clinical (Faber et al., 2005) Choonara and Sammons (2014) such as caffeine and midazolam, which are also used in pediatrics. Antibiotics are commonly used in pediatrics and many of them are eliminated by glomerular filtration. Thus, the probe drugs selected in our study can generally also represent pediatric medication eliminated by renal-filtration, CYP1A2 and CYP3A4 metabolism pathway. The probe drug models were verified in Caucasian adult population, Caucasian pediatric population, and Chinese adult population models. The accuracy of some certain simulations reduced due to the character of participants like pathological status, smoking history, or preterm birth.

**FIGURE 5:**
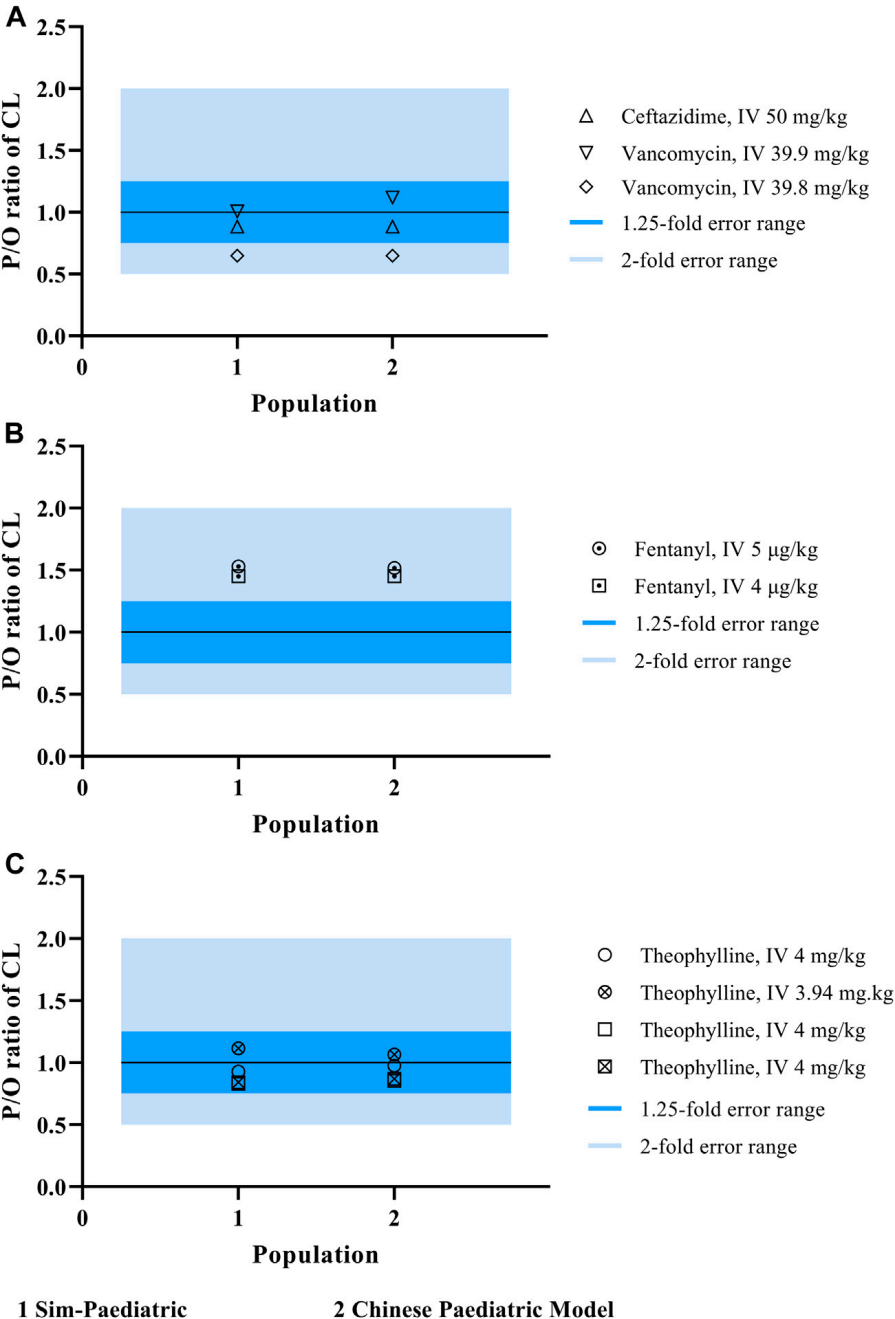
The P/O ratio of CL simulated by both NEC pediatric population model and Chinese pediatric population model.

Our population model was developed based on the inbuilt NEC pediatric population model in Simcyp, with height, weight, cardiac output, and tissue volumes (brain volume, heart volume, kidney volume, liver volume, pancreas volume, and spleen volume) recalibrated with Chinese pediatrics data. To confirm whether there was an ethnic difference between Chinese children and Caucasian children, we performed the same simulation using the NEC pediatric population model. However, the Chinese pediatric model showed no significant improvement in predicting the CL of CYP3A4-metabolized and renal-eliminated drugs, as shown in Figures 5A,B. This may be a system deviation resulted from the limited PK data. Figure 5C showed that the Chinese pediatric population model had a better estimation than the NEC pediatric model in CL prediction, after modification.

Although great efforts have been made to search for physiological data extensively in Chinese pediatric patients, there is still a lack of information on many physiological parameters such as body surface area (BSA), glomerular filtration rate (GFR), and especially on the ontogeny of a cytochrome P450 enzyme. Microsomal protein per gram of liver (MPPGL) and CYP enzyme ontogeny is critical in order to correctly predict the exposure of drug with metabolism as the major elimination pathway. Hence in our study, we assumed the Chinese pediatric population shares the same CYP1A2 and CYP3A4 ontogeny as the Caucasian population, which may not necessarily true. More clinical trials need to be carried out in order to prove our assumption. In addition, all the PK data of the Chinese pediatric population were from patients. Pathological factors would influence the accuracy of a prediction.

## Conclusion

In this study, a preliminarily physiologically based pharmacokinetic model based on Chinese pediatric physiological characteristics was developed. The model was verified in the Chinese pediatric population by predicting PK behavior of theophylline, fentanyl, ceftazidime, and vancomycin which are eliminated either through CYP1A2, CYP3A4 and renal pathway. In comparison of the observed PK data, our model successfully predicted the PK profile of these drugs within a 2-fold error range. However, it warrants further study to improve this population model.

## Data Availability

The original contributions presented in the study are included in the article/Supplementary Material, further inquiries can be directed to the corresponding author.
