# Supplementary material for: Development of a Virtual Chinese Pediatric Population Physiological Model Targeting Specific Metabolism and Kidney Elimination Pathways
Source: Front Pharmacol. 2021 May 11;12:648697. doi: 10.3389/fphar.2021.648697 (PMC8145459; doi:10.3389/fphar.2021.648697)
Supplement: Supplementary file 1 [file DataSheet1.docx]

**Supplementary Information Titles**

**Table S1. The sources of physiological parameters**

| Reference | Age range | Sex (M/F) | Sample size | Systematic parameter |
| --- | --- | --- | --- | --- |
| (1) | 0 | M | 481 | height, weight |
| (1) | 0 | F | 487 | height, weight |
| (2) | 0-18 | M | NA | height, weight |
| (2) | 0-18 | F | NA | height, weight |
| (3) | 0-15 | M & F | NA | brain weight, heart weight, liver weight, kidney weight, pancreas weight, spleen weight |
| (4) | 0-15 | M & F | 1000 | brain weight, heart weight, liver weight, kidney weight, pancreas weight, spleen weight |
| (5) | 0-18 | M & F | 1651 | cardiac output |

**Table S2. Probe drug PBPK model verification in different populations^a^**

| CYP1A2 pathway | Population | Reference | Sample size | C_max_ (mg·L^-1^) | | | CL (mL·h^-1^·kg^-1^) | | | AUC (mg·L^-1^·h) | | |
| --- | --- | --- | --- | --- | --- | --- | --- | --- | --- | --- | --- | --- |
|  |  |  |  | Model | Observed | Ratio | Model | Observed | Ratio | Model | Observed | Ratio |
| Theophylline | Chinese adult | (6) | 8 | 4.80±1.68 | 6.13±1.06 | 0.78 | 91.65±42.57 | 56.00±12.00 | 1.64 | 32.39±10.52 | 68.76±22.42 | 0.47 |
|  |  | (7) | 6 | 4.84±1.53 | 6.19±1.01 | 0.78 | 92.98±35.95 | 46.80±4.80 | 1.99 | 32.12±9.71 | 84.32±15.76 | 0.38 |
|  |  | (8) | 12 | 3.00±1.05 | 1.88±0.15 | 1.60 | 53.95±31.04 | NA | NA | 35.57±12.61 | 22.97±1.47 | 1.55 |
|  | Caucasian adult | (9) | 8 | 3.17±1.10 | 4.10 | 0.77 | 45.69±22.57 | 43.00 | 1.06 | 45.59±18.91 | 52.00 | 0.88 |
|  |  |  | 8 | 6.36±2.21 | 8.00 | 0.80 | 45.27±22.43 | 41.00 | 1.10 | 91.95±38.12 | 106.00 | 0.87 |
|  |  |  | 8 | 9.56±3.34 | 10.50 | 0.91 | 45.00±22.15 | 42.00 | 1.07 | 139.08±57.61 | 161.00 | 0.86 |
|  |  |  | 8 | 12.78±4.47 | 15.10 | 0.85 | 44.59±22.02 | 41.00 | 1.09 | 186.95±77.38 | 210.00 | 0.89 |
|  | Caucasian children | (10) | 30 | 7.09±1.85 | NA | NA | 94.67±38.66 | 87.00±35.00 | 1.09 | 35.02±8.94 | NA | NA |

| CYP3A4 pathway | Population | Reference | Sample size | C_max_ (ng·mL^-1^) | | | CL (mL·min^-1^·kg^-1^) | | | AUC (ng·mL^-1^·h) | | |
| --- | --- | --- | --- | --- | --- | --- | --- | --- | --- | --- | --- | --- |
|  |  |  |  | Model | Observed | Ratio | Model | Observed | Ratio | Model | Observed | Ratio |
| Fentanyl | Chinese adult | (11) | 8 | 34.92±2.70 | NA | NA | 11.99±2.43 | 13.78±3.83 | 0.87 | 7.17±1.49 | NA | NA |
|  |  | (12) | 8 | 62.85±4.65 | 36.77±8.24 | 1.71 | 13.58±2.44 | 17.05±4.78 | 0.80 | 25.12±4.37 | 20.58±4.13 | 1.22 |
|  |  |  | 8 | 62.10±5.04 | 47.14±20.97 | 1.32 | 13.66±2.44 | 11.25±5.85 | 1.21 | 24.95±4.39 | 34.36±11.03 | 0.73 |
|  |  | (13) | 10 | 36.40±2.40 | 13.4 | 2.72 | 18.79±2.92 | 12.61±4.32 | 1.49 | 4.49±0.65 | NA | NA |
|  |  | (14) | 22 | 28.73±2.24 | NA | NA | 16.90±2.52 | 13.79±3.84 | 1.23 | 3.99±0.62 | NA | NA |
|  | Caucasian adult | (15) | 13 | 8.74±0.97 | 3.60±1.10 | 2.43 | 12.41±2.77 | 15.30±5.00 | 0.81 | 6.88±1.45 | 5.08±1.51 | 1.35 |
|  |  | (16) | 12 | 36.84±4.64 | NA | NA | 13.45±2.83 | 15.60±8.20 | 0.86 | 6.31±1.29 | 4.80±2.70 | 1.31 |
|  |  | (17) | 12 | 0.53±0.15 | 0.21±0.10 | 2.52 | 36.91±14.80 | NA | NA | 1.26±0.46 | 0.68±0.32 | 1.85 |
|  |  | (18) | 7 | 155.27±18.49 | 93.98 | 1.65 | 18.80±2.98 | NA | NA | 18.39±2.90 | 18.87^b^ | 0.97 |
|  |  | (19) | 16 | 2.64±0.31 | 2.07 | 1.28 | 14.10±2.86 | NA | NA | 3.01±0.60 | 2.90^b^ | 1.04 |
|  |  | (20) | 11 | 11.08±1.23 | 4.37±2.46 | 2.53 | 11.77±2.66 | 18.80±8.20 | 0.63 | 8.30±1.91 | 5.48±2.93 | 1.51 |
|  |  |  | 5 | 11.11±1.21 | 4.24±1.45 | 2.62 | 11.57±2.60 | 19.50±1.80 | 0.59 | 8.43±1.86 | 4.31±0.44 | 1.96 |
|  |  | (21) | 10 | 20.81±2.28 | NA | NA | 12.91±2.96 | 23.90±9.90 | 0.54 | 3.97±0.87 | NA | NA |
|  | Caucasian children | (18) | 7 | 116.19±5.51 | 91.54 | 1.27 | 20.27±4.32 | NA | NA | 25.85±2.55 | 16.14 | 1.60 |
|  |  |  | 7 | 117.40±10.26 | 154.31 | 0.76 | 23.25±6.29 | NA | NA | 22.11±2.82 | 25.10 | 0.88 |
|  |  | (22) | 14 | 198.76±10.88 | NA | NA | 12.36±2.23 | 22.41±8.20 | 0.55 | 71.33±6.24 | NA | NA |

| Renal filtration | Population | Reference | Sample size | C_max_ (mg·L^-1^) | | | CL(mL·h^-1^·kg^-1^) | | | | | AUC (mg·L^-1^·h) | | |
| --- | --- | --- | --- | --- | --- | --- | --- | --- | --- | --- | --- | --- | --- | --- |
|  |  |  |  | Model | Observed | Ratio | Model | | Observed | Ratio | Model | | Observed | Ratio |
| Vancomycin | Chinese adult | (23) | 10 | 30.88±2.97 | 46.31±4.48 | 0.67 | 86.13±14.51 | | 75.96±8.22 | 1.14 | 177.84±28.39 | | 192.78±20.23 | 0.92 |
|  | Caucasian adult | (24) | 4 | 45.11±5.29 | NA | NA | 104.29±22.22 | | 65.10±4.26 | 1.60 | 141.81±29.42 | | NA | NA |
|  |  | (25) | 4 | 22.64±3.21 | NA | NA | 78.43±15.90 | | 71.40±0.79 | 1.10 | 86.84±15.53 | | NA | NA |
|  |  | (26) | 10 | 27.56±2.66 | NA | NA | 82.49±18.55 | | 68.29±16.88 | 1.21 | 131.63±28.73 | | 135.00±53.00 | 0.98 |
|  | Caucasian children | (27) | 15 | 34.15±1.06 | 31.20±12.00 | 1.09 | | 82.89±37.43 | 68.48±39.29 | 1.21 | 169.04±45.09 | | NA | NA |
|  |  | (28) | 4 | 22.71±1.73 | NA | NA | | 128.55±62.04 | 73.54±43.65 | 1.75 | 84.21±25.65 | | NA | NA |
|  |  | (29) | 6 | 115.49±8.76 | 46.63 | 2.48 | | 130.36±59.45 | 120.00±20.00 | 1.09 | 423.44±125.93 | | NA | NA |
|  |  | (30) | 374 | 36.14±2.17 | NA | NA | | 128.86±58.08 | 66.00 | 1.95 | 127.74±30.36 | | NA | NA |
| Ceftazidime | Chinese adult | (31) | 6 | 82.14±9.00 | 114.30±33.54 | 0.72 | | 102.27±16.16 | 83.32±19.84 | 1.23 | 152.64±22.33 | | 207.83±61.72 | 0.73 |
|  | Caucasian adult | (32) | 6 | 79.46±7.81 | 72.10±3.60 | 1.10 | | 86.99±12.50 | NA | NA | 148.22±21.44 | | 143.20±4.50 | 1.04 |
|  |  |  | 8 | 108.68±10.78 | 119.10±9.50 | 0.91 | | 87.50±12.75 | NA | NA | 147.32±21.45 | | 135.80±9.50 | 1.09 |
|  |  |  | 8 | 54.41±5.35 | 57.6±3.80 | 0.94 | | 87.50±12.63 | NA | NA | 73.66±10.72 | | 71.90±1.80 | 1.02 |
|  |  |  | 7 | 158.90±15.62 | 170.00±6.80 | 0.93 | | 86.99±12.50 | NA | NA | 296.47±42.90 | | 266.00±8.40 | 1.12 |
|  |  |  | 8 | 209.47±19.58 | 182.80±16.30 | 1.15 | | 86.87±12.63 | NA | NA | 296.62±43.03 | | 279.40±8.50 | 1.06 |
|  |  | (33) | 6 | 144.07±15.51 | 159.30±55.20 | 0.90 | | 90.72±13.49 | 81.44±8.91 | 1.11 | 286.58±42.09 | | 287.00±56.00 | 1.00 |
|  |  | (34) | 4 | 53.86±5.14 | 65.80 | 0.82 | | 86.79±13.35 | 93.24 | 0.93 | 74.24±11.05 | | 71.40 | 1.04 |
|  |  | (35) | 7 | 213.74±22.57 | 210.00 | 1.02 | | 90.98±13.45 | 98.36 | 0.92 | 285.20±42.46 | | NA | NA |
|  |  | (36) | 6 | 142.49±15.14 | NA | NA | | 89.34±13.70 | 197.14±57.14 | 0.45 | 287.62±42.63 | | 156.30±51.10 | 1.84 |
|  |  | (37) | 9 | 214.81±21.03 | NA | NA | | 89.02±13.88 | NA | NA | 292.69±44.31 | | 276.70±9.80 | 1.06 |
|  | Caucasian children | (38) | 30 | 282.44±14.92 | 77.00±8.00 | 3.67 | | 76.56±14.35 | 50.40±4.80 | 1.52 | 343.15±65.86 | | NA | NA |

NA not applicable

^a^ PK parameters expressed as mean (±SD).

^b^ Calculated using trapezoidal rule from concentration-time profiles in literature.


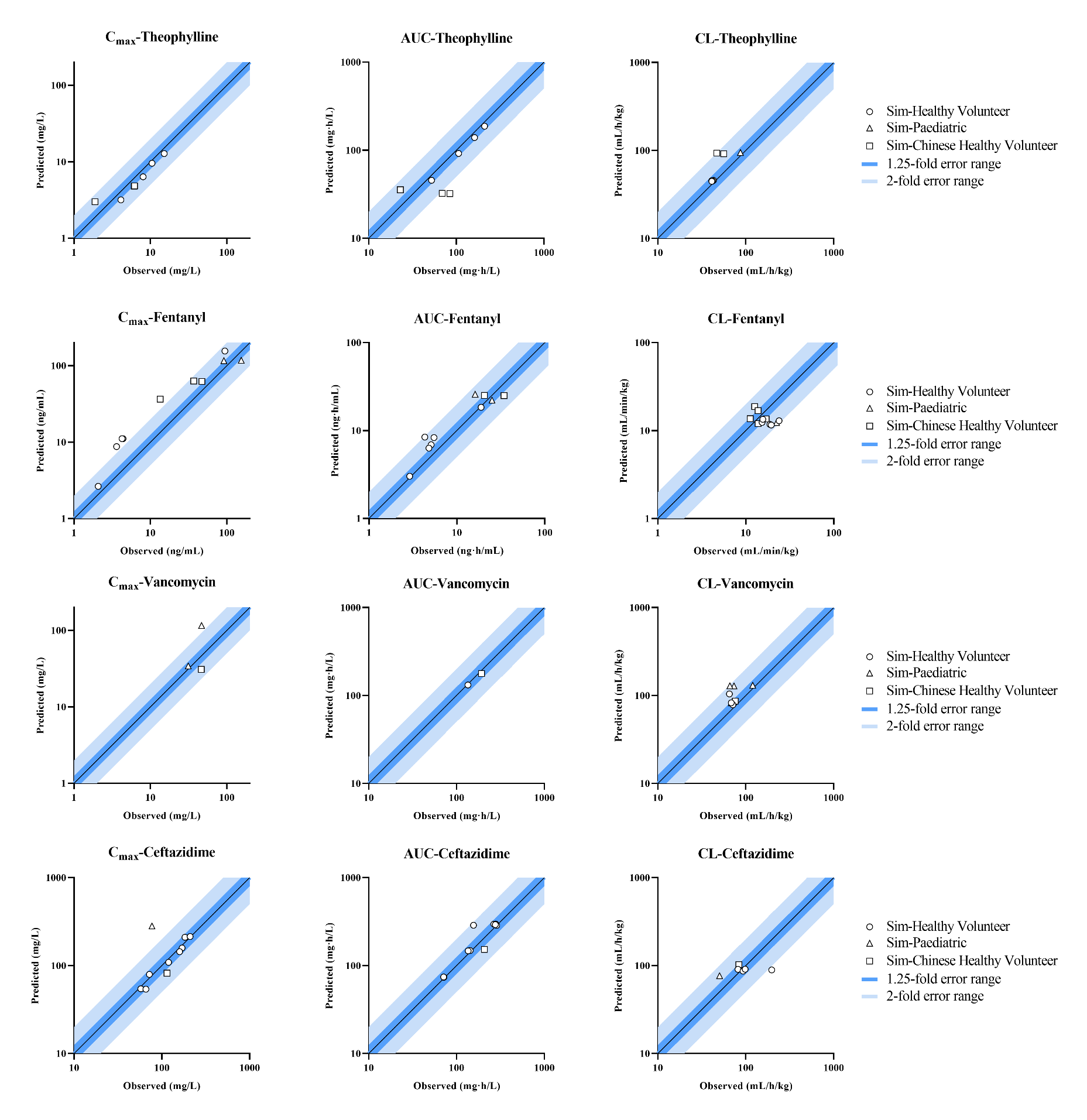


**Fig. S1** The observed PK parameter (C_max_, CL, AUC) versus predicted values in probe drugs PBPK model verification.


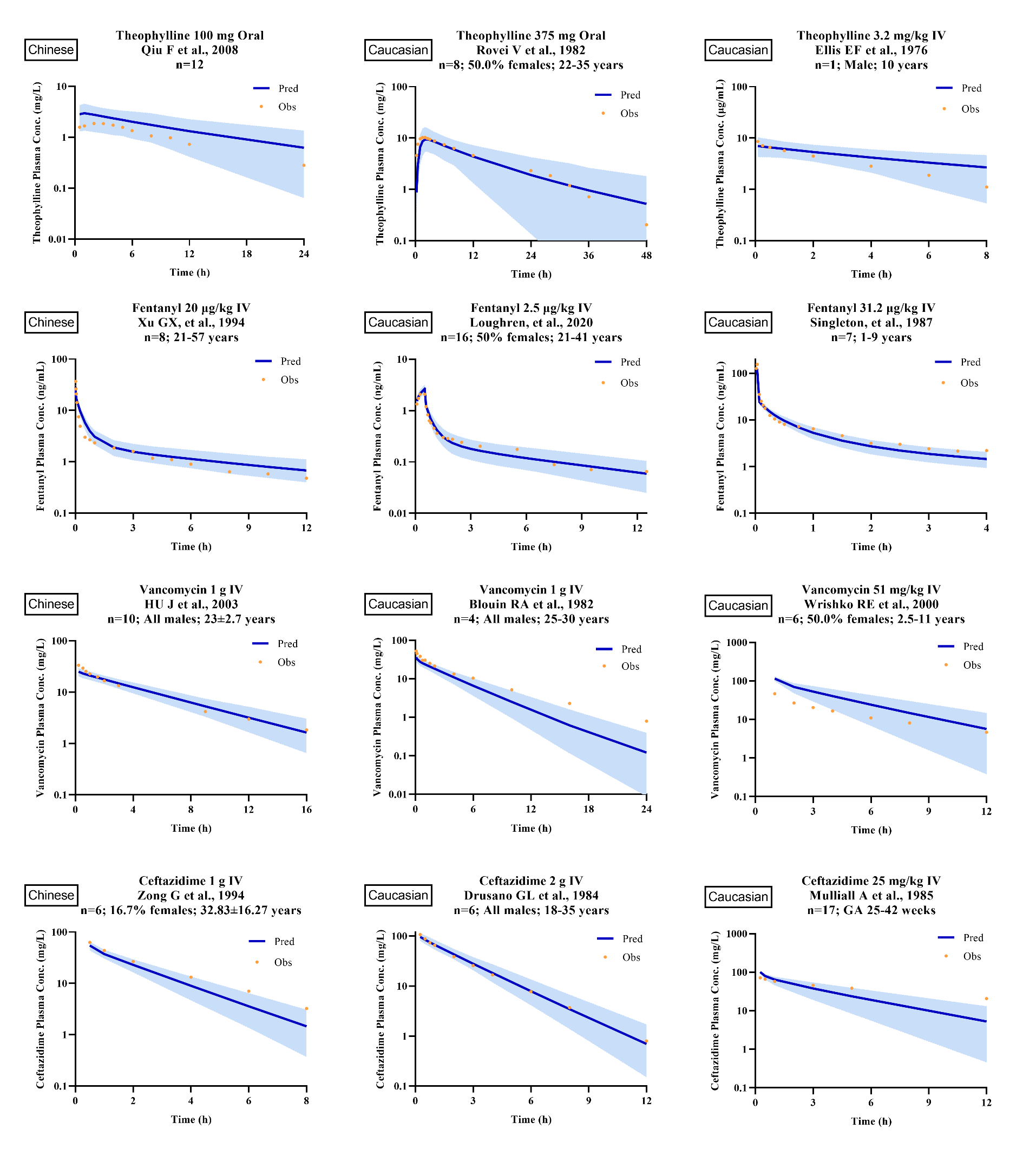


**Fig. S2** The observed and predicted concentration data of probe drugs in drug models verification. (Dots represents observed data; blue line represents predicted data; and blue shadow represents 90% prediction interval)

Reference:

1. Janssen PA, Thiessen P, Klein MC, Whitfield MF, Macnab YC, Cullis-Kuhl SC. Standards for the measurement of birth weight, length and head circumference at term in neonates of European, Chinese and South Asian ancestry. *Open Med* (2007) 1(2):e74-e88. PubMed PMID: 20101298.

2. Zong X-N, Li H. Construction of a new growth references for China based on urban Chinese children: comparison with the WHO growth standards. *PloS one* (2013) 8(3):e59569-e. Epub 2013/03/19. doi: 10.1371/journal.pone.0059569. PubMed PMID: 23527219.

3. Wang J, Li B, Chen R, Zhao Y, Xu Y, Gao Z, et al. Reference values of main internal organs for Chinese. *Chinese Journal of Radiological Medicine and Protection* (1995) 15(4):248-54. PubMed PMID: 282899.

4. Kawamura H. Organ mass measurements. International Atomic Energy Agency (IAEA): (1998) 1011-4289 Contract No.: IAEA-TECDOC--1005(v1).

5. Cattermole GN, Leung PY, Ho GY, Lau PW, Chan CP, Chan SS, et al. The normal ranges of cardiovascular parameters measured using the ultrasonic cardiac output monitor. *Physiol Rep* (2017) 5(6). Epub 2017/03/23. doi: 10.14814/phy2.13195. PubMed PMID: 28320891; PubMed Central PMCID: PMCPMC5371563.

6. Li YL, Zheng QK, Zhang X. Effect of levofloxacin on pharmacokinetics of theophylline in healthy volunteers and chronic obstructive pulmonary disease patients [J]. *Chinese Journal of Clinical Pharmacy* (1999) 4.

7. Li YL, Zheng QK. The effect of ofloxacin on pharmacokinetic of theophylline in healthy subjects [J]. *CHINESE JOURNAL OF HOSPITAL PHARMACY* (1997) 9.

8. Qiu F, Wang G, Zhao Y, Sun H, Mao G, A J, et al. Effect of danshen extract on pharmacokinetics of theophylline in healthy volunteers. *British Journal of Clinical Pharmacology* (2008) 65(2):270-4. doi: 10.1111/j.1365-2125.2007.03027.x.

9. Rovei V, Chanoine F, Benedetti SM. Pharmacokinetics of theophylline: a dose-range study. (1982) 14(6):769-78. doi: 10.1111/j.1365-2125.1982.tb02035.x.

10. Ellis EF, Koysooko R, Levy G. Pharmacokinetics of Theophylline in Children With Asthma. *Pediatrics* (1976) 58(4):542.

11. Xu K, Yue J, Chen S, Guo P, Yang Z. Pharmacokinetics of fentanyl in patients of different ages undergoing non-cardiac surgery. *Chin J Anes thesiol* (2001) 21(1).

12. Xu G, Yin W, Liu L, Yin D. Pharmacokinetics of fentanyl in Chinese patients during operation. *Chin J Clin Pharmacol* (1994) 10(3):157~64.

13. Xu K, Li Y, Yue J. The pharmacokinetics of domestic fentanyl in patients undergoing orthopaedic surgery. *J North China Coal Medical College* (2005) 7(6):698-700. doi: 10.19539/j.cnki.

14. Li J, Xu d, Gao X. Comparative study of the pharmacokinetics of fentanyl citrate spinal anesthesia in patients with different ages. *CHINA HEALTH CARE ＆ NUTRITION* (2013):548-9.

15. Ibrahim AE, Feldman J, Karim A, Kharasch ED. Simultaneous Assessment of Drug Interactions with Low- and High-Extraction Opioids. *Anesthesiology* (2003) 98(4):853-61. doi: 10.1097/00000542-200304000-00011.

16. Olkkola KT, Palkama VJ, Neuvonen PJ. Ritonavir's Role in Reducing Fentanyl Clearance and Prolonging Its Half-life. *Anesthesiology* (1999) 91(3):681-. doi: 10.1097/00000542-199909000-00020.

17. Kharasch ED, Hoffer C, Altuntas TG, Whittington D. Quinidine as a Probe for the Role of P-Glycoprotein in the Intestinal Absorption and Clinical Effects of Fentanyl. *The Journal of Clinical Pharmacology* (2004) 44(3):224-33. doi: 10.1177/0091270003262075.

18. Singleton MA, Rosen JI, Fisher DM. Plasma concentrations of fentanyl in infants, children and adults. *Canadian Journal of Anaesthesia* (1987) 34(2):152-5. doi: 10.1007/bf03015333.

19. Loughren MJ, Kharasch ED, Kelton-Rehkopf MC, Syrjala KL, Shen DD. Influence of St. John’s Wort on Intravenous Fentanyl Pharmacokinetics, Pharmacodynamics, and Clinical Effects. *Anesthesiology* (2020) 132(3):491-503. doi: 10.1097/aln.0000000000003065.

20. Ziesenitz VC, König SK, Mahlke N, Jantos R, Skopp G, Weiss J, et al. Fentanyl Pharmacokinetics is not Dependent on Hepatic Uptake by Organic Anion-Transporting Polypeptide 1B1 in Human Beings. *Basic & Clinical Pharmacology & Toxicology* (2013) 113(1):43-8. doi: 10.1111/bcpt.12066.

21. Palkama VJ, Neuvonen PJ, Olkkola KT. The CYP 3A4 inhibitor itraconazole has no effect on the pharmacokinetics of i.v. fentanyl. *British Journal of Anaesthesia* (1998) 81(4):598-600. doi: 10.1093/bja/81.4.598.

22. Gauntlett IS, Fisher DM, Hertzka RE, Kuhis E, Spellman MJ, Rudolph C. Pharmacokinetics of Fentanyl in Neonatal Humans and Lambs. *Anesthesiology* (1988) 69(5):683-7. doi: 10.1097/00000542-198811000-00008.

23. Hu J, Shi Y, Zahng J. Pharmacokinetics of vancomycin in elderly and young healthy volunteers [J]. *Chinese Journal of Infection and Chemotherapy* (2003) 3.

24. Blouin RA, Bauer LA, Miller DD, Record KE, Griffen WO. Vancomycin pharmacokinetics in normal and morbidly obese subjects. *Antimicrobial agents and chemotherapy* (1982) 21(4):575-80.

25. Krogstad DJ, Moellering JR RC, Greenrlatt DJ. Single‐dose kinetics of intravenous vancomycin. *The Journal of Clinical Pharmacology* (1980) 20(4):197-201.

26. Rodvold KA, Blum RA, Fischer JH, Zokufa HZ, Rotschafer JC, Crossley KB, et al. Vancomycin pharmacokinetics in patients with various degrees of renal function. *Antimicrobial Agents and Chemotherapy* (1988) 32(6):848-52.

27. Reed MD, Kliegman RM, Weiner JS, Huang M, Yamashita TS, Blumer JL. The clinical pharmacology of vancomycin in seriously ill preterm infants. *Pediatric research* (1987) 22(3):360.

28. Alves GCdS, Chequer FMD, Sanches C. Effective vancomycin concentrations in children: a cross-sectional study. *Einstein (São Paulo)* (2019) 17(1).

29. Wrishko RE, Levine M, Khoo D, Abbott P, Hamilton D. Vancomycin pharmacokinetics and Bayesian estimation in pediatric patients. *Therapeutic drug monitoring* (2000) 22(5):522-31.

30. Capparelli EV, Lane FR, Romanowski GL, McFeely EJ, Murray W, Sousa P, et al. The influences of renal function and maturation on vancomycin elimination in newborns and infants. *The Journal of Clinical Pharmacology* (2001) 41(9):927-34.

31. Zong G, Xiao G, Zhang Y. The pharmacokinetics of ceftazidime in the burned patients. *Zhonghua zheng xing shao shang wai ke za zhi= Zhonghua zheng xing shao shang waikf [ie waike] zazhi= Chinese journal of plastic surgery and burns* (1994) 10(5):385-8.

32. Harding SM, Harper PB. The pharmacokinetic behaviour of ceftazidime in man and the relationship between serum levels and the in vitro susceptibility of clinical isolates. *Infection* (1983) 11(1):S49-S53. doi: 10.1007/BF01641107.

33. Drusano GL, Standiford HC, Fitzpatrick B, Leslie J, Tangtatsawasdi P, Ryan P, et al. Comparison of the pharmacokinetics of ceftazidime and moxalactam and their microbiological correlates in volunteers. *Antimicrobial Agents and Chemotherapy* (1984) 26(3):388. doi: 10.1128/AAC.26.3.388.

34. Harding SM, Monro AJ, Thornton JE, Ayrton J, Hogg MIJ. The comparative pharmacokinetics of ceftazidime and cefotaxime in healthy volunteers. *Journal of Antimicrobial Chemotherapy* (1981) 8(suppl_B):263-72. doi: 10.1093/jac/8.suppl_B.263.

35. Bulitta JB, Landersdorfer CB, Hüttner SJ, Drusano GL, Kinzig M, Holzgrabe U, et al. Population pharmacokinetic comparison and pharmacodynamic breakpoints of ceftazidime in cystic fibrosis patients and healthy volunteers. *Antimicrobial agents and chemotherapy* (2010) 54(3):1275-82. Epub 2010/01/11. doi: 10.1128/AAC.00936-09. PubMed PMID: 20065059.

36. Kalman D, Barriere SL, Johnson BL, Jr. Pharmacokinetic disposition and bactericidal activities of cefepime, ceftazidime, and cefoperazone in serum and blister fluid. *Antimicrobial agents and chemotherapy* (1992) 36(2):453-7. doi: 10.1128/aac.36.2.453. PubMed PMID: 1605609.

37. Ljungberg B, Nilsson-Ehle I. Pharmacokinetics of ceftazidime in elderly patients and young volunteers. *Scand J Infect Dis* (1984) 16(3):325-6. Epub 1984/01/01. doi: 10.3109/00365548409070410. PubMed PMID: 6387894.

38. Mulliall A, Louvois Jd. The pharmacokinetics and safety of ceftazidiine in the neonate. *Journal of Antimicrobial Chemotherapy* (1985) 15(1):97-103. doi: 10.1093/jac/15.1.97.
